# Association of Legal Intervention Injuries With Race and Ethnicity Among Patients Treated in Emergency Departments in California

**DOI:** 10.1001/jamanetworkopen.2018.2150

**Published:** 2018-09-14

**Authors:** Alyssa C. Mooney, Shannon McConville, Aaron J. Rappaport, Renee Y. Hsia

**Affiliations:** 1 Department of Epidemiology and Biostatistics, University of California, San Francisco; 2 Public Policy Institute of California, San Francisco; 3 Hastings College of the Law, University of California, San Francisco; 4 Department of Emergency Medicine, University of California, San Francisco; 5 Philip R. Lee Institute for Health Policy Studies, University of California, San Francisco

## Abstract

**Importance:**

Increased public concern regarding police use of force has coincided with a dearth of available data to uncover the magnitude and trends in injuries, particularly across race or ethnicity.

**Objective:**

To examine trends in injury rates, severity, and disparities across black individuals, white individuals, Hispanic individuals, and Asian/Pacific Islander individuals.

**Design, Setting, and Participants:**

In this retrospective, cross-sectional study, data collected on every hospital visit in California from January 1, 2005, to September 30, 2015, were used to model trends in rates of legal intervention injuries (n = 92 386) per capita and per arrest for men aged 14 to 64 years, by race or ethnicity. The study also examined descriptive statistics on injury dispositions to assess changes in severity. Analyses were conducted between December 2017 and June 2018.

**Main Outcomes and Measures:**

All visits with an external cause of injury code of E970 to E977 were classified as legal intervention injuries. This range of codes includes injuries inflicted by the police or other law-enforcing agents in the course of arresting or attempting to arrest lawbreakers, suppressing disturbances, maintaining order, and other legal action.

**Results:**

The study identified a total of 92 386 hospital visits that were the result of legal intervention among males aged 14 to 64 years. Black individuals were at the highest risk of legal intervention injury per capita in 2005 (for black vs white individuals, rate ratio, 2.90; 95% CI, 2.74-3.06), and remained so across the study period. Although rates among Asian/Pacific Islander individuals remained stable, rates in all other groups increased from 2005 to 2009 and then declined from 2009 to 2015, nearly returning to 2005 levels. During the period of increasing rates, the black to white disparity widened by 3% annually (rate ratio, 1.03; 95% CI, 1.01-1.05), then narrowed as rates declined. In contrast, rates of injury per arrest have increased over the past decade, although rates were broadly similar across race or ethnicity. The proportion of injuries involving firearms (ie, shootings by police) declined from 7.0% in 2005 and 2006 to 3.7% in 2014 and 2015.

**Conclusions and Relevance:**

States with central repositories for hospital visits offer data sources to illuminate the public health problem of legal intervention injuries, and warrant greater attention to ensure consistent coding for complete capture.

## Key Points

**Question:**

What were the trends in legal intervention injuries from 2005 to 2015 in California?

**Findings:**

A cross-sectional analysis of all hospital visits in California indicated that rates of legal intervention injuries per capita increased until 2009 and were in decline through 2015. Black individuals were consistently at greatest risk of legal intervention injury per capita, although rates per arrest were broadly similar across race and ethnicity. Injuries involving firearms declined.

**Meaning:**

Hospital data provide an opportunity to monitor the magnitude and characteristics of legal intervention injuries, although efforts are needed to ensure complete reporting on this public health problem disproportionately associated with communities of color.

## Teaser

This cross-sectional study examines all outpatient emergency department (ED) visits and inpatient admissions for injuries following legal interventions by police and other law enforcement agents and trends in injury rates and severity and association with disparities by race and ethnicity of the treated patients.

## Introduction

Public concern about police use of force has intensified in recent years following a series of highly publicized shootings of unarmed black men.^1,2,3,4,5,6,7^ However, efforts to assess disparities in the use of force and changes over time have faced a basic obstacle: the lack of reliable data regarding the prevalence of law enforcement–related injuries.^8,9^

Numerous governmental databases aspire to measure deaths due to police action, but all have significant and well-known methodological problems. For example, the Federal Bureau of Investigation’s Supplementary Homicide Reports and the Bureau of Justice Statistics’ Arrest-Related Deaths Program are both voluntary reporting systems with uneven compliance; the National Vital Statistics System, developed by the National Center for Health Statistics, relies on reports by death certifiers, who often fail to identify police involvement in homicides.^10,11,12^ Victim surveys by the US Department of Justice have additional shortcomings, such as the lack of information on the location of interactions, inaccuracies associated with self-reporting, the exclusion of incarcerated individuals, and infrequent implementation (once every 3 years).^13^

With no comprehensive national data on fatal injuries inflicted by police, *The Guardian* developed The Counted,^14^ which uses reporting and crowdsourced information to build a database of deaths inflicted by police or other law enforcement agencies. However, nonfatal injuries are excluded. Some public health researchers have looked to alternative measures of police-related injuries to address the gap. One source has been national samples of emergency department (ED) visits, which include codes for legal intervention. Among these are the Centers for Disease Control and Prevention’s Web-Based Injury Statistics Query and Reporting System, the National Inpatient Sample, and the Nationwide Emergency Department Sample. The latter 2 are part of the Healthcare Cost and Utilization Project family of databases.^15,16^ Several recent studies have used these data sources to examine police use of force injuries,^17,18,19^ although none of these studies have examined trends in injuries by race or ethnicity.

To address these shortcomings, we used a new data source—information collected on every outpatient ED visit and inpatient admission in California during an 11-year period (2005-2014). Because California has a central repository for these data and requires all hospitals to collect patient race or ethnicity, the analysis offers insights into trends among white individuals, black individuals, Hispanic individuals, and Asian/Pacific Islander individuals not possible with other data sets. The data provide a unique opportunity for longitudinal analysis of all legal intervention injuries passing through any hospital, including the primary reason for the visit, any additional diagnoses recorded during the visits, the injury mechanism, and patient demographic characteristics.

## Methods

Nonpublic (restricted use) patient discharge data and ED data were obtained from the California Office of Statewide Hospital Planning and Development. All licensed hospitals in the state except those that are federally operated are required to submit information on all inpatient and ED visits. We performed a retrospective, cross-sectional analysis of all outpatient ED visits and inpatient admissions for legal intervention injuries among males aged 14 to 64 years in California from January 1, 2005, to September 30, 2015, after which the *International Classification of Diseases, Ninth Revision, Clinical Modification* (*ICD-9-CM*) coding system shifted to *International Classification of Diseases, Tenth Revision, Clinical Modification *(*ICD-10-CM*). We anticipated a period of unreliable and noncomparable coding during the last 3 months of 2015, so they were excluded from the analysis. For tables and figures displaying 2015 data, we used the model of secular and seasonal trends beginning in 2005 to project injury counts for the last 3 months of 2015. The analysis was restricted to males, as this group makes up 89% of legal intervention injuries. We followed the Strengthening the Reporting of Observational Studies in Epidemiology (STROBE) reporting guideline. The University of California, San Francisco’s institutional review board approved this study. Participant consent was waived because the researchers did not know the identity of the study participants.

### Measures

To identify injuries that were the result of law enforcement contact, we used external cause of injury codes (E-codes) described in the *ICD-9-CM* system.^20^ E-codes are intended to capture how an injury occurred and the intent. For each case, the hospital discharge data contain a primary E-code and up to 4 additional E-codes. All cases with an E-code of E970 to E977 were classified as legal intervention injuries. This range of codes includes injuries inflicted by the police or other law-enforcing agents in the course of suppressing disturbances, maintaining order, arresting or attempting to arrest offenders, or other legal action.

### Statistical Analysis

We present statewide summaries of demographic characteristics of patients with legal intervention injuries, and the mechanisms, dispositions, primary diagnoses, and co-occurring diagnoses for those visits. We then model trends in monthly rates of legal intervention injuries by race or ethnicity (non-Hispanic white individuals, black individuals, Hispanic individuals, and Asian/Pacific Islander individuals), per population and per arrest. Poisson models were specified with robust standard errors and offsets for race-specific population and arrest counts to generate rates per population and per arrest, respectively. Calendar month dummies controlled for seasonal trends, and secular trends were modeled using linear splines for month-year to allow for changes in trend. Spline knots were placed at 2009 for the injuries per population model and 2013 for the injuries per arrest model, selected based on visual inspections of graphs of monthly rates to identify the points at which trends shifted. Secular trend variables were interacted with race or ethnicity to allow trends to differ by group. Linear combinations of model coefficients for trends in monthly rates were used to generate estimates of annual changes in rates and rate ratios (RRs) for given time periods, and 95% confidence intervals were calculated.

Figures display crude rates of visits per population and per arrest. We used the model of secular and seasonal trends to project injury counts for the last 3 months of 2015, during which *ICD* coding shifted to *ICD-10-CM*. Annual population estimates were from the National Cancer Institute’s Surveillance, Epidemiology, and End Results Program.^21^ Counts of total annual arrests were from the Monthly Arrests and Citations Register from the California Department of Justice.^22^ Analyses were conducted in 2018, using Stata, version 15 (StataCorp LP).

## Results

### Characteristics of ED Patients With Legal Intervention Injuries

Over the 11-year study period, we identified a total of 92 386 hospital visits that were the result of legal intervention among males aged 14 to 64 years (Table 1). Compared with their representation in the population, injuries were disproportionately high among younger age groups, black individuals, and Hispanic individuals. Men aged 25 to 34 represented 31.8% of injuries (n = 29 404), but 21.1% of the population (n = 2 700 529). Black individuals made up 18.5% of injuries (n = 17 132), but just 6.6% of the population (n = 839 550). Hispanic individuals made up 39.4% of injuries (n = 36 384) and 36.7% of the population (n = 4 683 825).

**Table 1.** Demographic Characteristics of Male Patients Aged 14 to 64 Years With 92 386 Legal Intervention Injuries From 2005 to 2015

Demographic Characteristics	No. (%)	
	Injuries Associated With Legal Intervention	Population
Age, y		
14-17	6870 (7.4)	1 086 647 (8.5)
18-24	21 304 (23.1)	1 969 169 (15.4)
25-34	29 404 (31.8)	2 700 529 (21.1)
35-44	19 432 (21.0)	2 584 303 (20.2)
45-54	11 769 (12.7)	2 520 988 (19.7)
55-64	3607 (3.9)	1 914 185 (15.0)
Race/ethnicity		
White	36 913 (40.0)	5 457 217 (42.7)
Black	17 132 (18.5)	839 550 (6.6)
Hispanic	36 384 (39.4)	4 683 825 (36.7)
Asian/Pacific Islander	1957 (2.1)	1 795 229 (14.1)

### Characteristics of Legal Intervention Injuries

Injury severity appears to have changed over time. Table 2 compares characteristics of injuries for 2005 and the first 9 months of 2006, with 2014 and the first 9 months of 2015, after which *ICD-9-CM* coding shifted to *ICD-10-CM*. Across these 2 periods, there was a substantial reduction in the proportion of legal intervention injuries involving firearms (ie, shootings by police) from 997 (7.0%) to 564 (3.7%), and the proportion of injuries resulting in death declined from 58 (0.4%) to 40 (0.3%). Together, these would suggest reductions in severity. However, the converse finding was an increasing proportion of injuries resulting in a hospital admission (621 [4.3%] to 864 [5.6%]) rather than outpatient visit (13 541 [94.5%] to 14 449 [93.5%]).

**Table 2.** Characteristics of Legal Intervention Injury Visits Among Males Aged 14 to 64 Years From 2005 to 2006 and 2014 to 2015

Injury Characteristic	No. (%)^a^		*P* Value^b^
	2005-2016 (n = 14 324)	2014-2015 (n = 15 458)	
Disposition, visit type			
Outpatient, ED	13 541 (94.5)	14 449 (93.5)	<.001
Inpatient, admitted through ED	621 (4.3)	864 (5.6)	<.001
Inpatient, not through ED	162 (1.1)	145 (0.94)	.10
Died	58 (0.4)	40 (0.3)	.03
Mechanism			
Blunt object/cutting	1902 (13.3)	1596 (10.3)	<.001
Firearms	997 (7.0)	564 (3.7)	<.001
Blow/manhandling	9943 (65.9)	11 497 (74.4)	<.001
Unspecified	1624 (11.3)	1264 (8.2)	<.001
Other	358 (2.5)	537 (3.5)	<.001
Primary diagnosis			
Contusions	5217 (36.4)	4466 (28.9)	<.001
Open wound, head	1921 (13.4)	1647 (10.6)	<.001
Medical examination	1107 (7.7)	1660 (10.7)	<.001
Sprains	1163 (8.1)	1147 (7.4)	.02
Other external cause	854 (6.0)	1391 (9.0)	<.001
Open wound, extremities	1045 (7.3)	1163 (7.5)	.45
Fracture, upper limb	455 (3.2)	446 (2.9)	.14
Any mental health diagnosis	1375 (9.6)	4086 (26.4)	<.001
Any mental health diagnosis, assaults not associated with legal intervention	12 574 (8.6)	37 037 (24.6)	<.001
Alcohol use disorder and/or SUD diagnosis	2232 (15.6)	3435 (22.2)	<.001
SUD diagnosis, assaults not associated with legal intervention^c^	19 164 (13.1)	28 079 (18.6)	<.001

Abbreviations: ED, emergency department; SUD, substance use disorder.

^a^ Each time period contains equal numbers of months. The 2006 data only include the first 9 months of the year for consistency with the 2015 data. These periods were created to account for the shift from *International Classification of Diseases, Ninth Revision, Clinical Modification *to *International Classification of Diseases, Ninth Revision, Clinical Modification *in October 2015, at which point injury codes may not be comparable with previous periods. Primary diagnosis is based on the Agency for Healthcare Research and Quality Clinical Classifications Software codes that were merged on to *International Classification of Diseases, Ninth Revision, Clinical Modification *codes, reported for the primary diagnosis.

^b^ *P* values are based on *t* tests of means between the 2 periods.

^c^ Mental health and SUD also used Clinical Classifications Software codes, but incorporated all available diagnoses including primary diagnosis and up to 24 other diagnosis recorded in the discharge abstract.

The data also indicate an increase in the incidence of a co-occurring behavioral health diagnosis for patients with legal intervention injuries. Legal intervention ED visits with any diagnosis indicating a mental health condition increased from 12 574 (8.6%) to 37 037 (24.6%). Visits with an alcohol- or substance-related disorder increased as well, from 2232 (15.6%) to 3435 (22.2%). Similar trends were seen in co-occurring diagnoses for patients with injuries from assaults not associated with legal intervention.

### Trends in Legal Intervention Injury Rates and Racial or Ethnic Disparities

**Figure 1.**
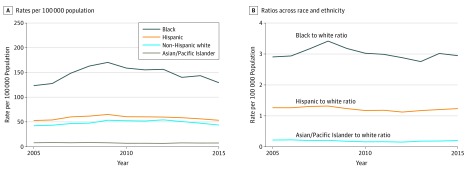
Rates of Legal Intervention Injuries per 100 000 Population and Ratios Across Race or Ethnicity of Males Aged 14 to 64 Years

Black individuals were at the greatest risk of legal intervention injury on a population level. In 2005, black individuals experienced 159 injuries per 100 000 population compared with 55 among white individuals, a 2.90-fold difference (95% CI, 2.74-3.06) (Table 3 and Figure 1). While rates among black individuals were highest to begin with, increases were also steepest in this population from 2005 to 2009. During this period, rates increased by 11% per year among black individuals (RR, 1.11; 95% CI, 1.09-1.13), 8% among white individuals (RR, 1.08; 95% CI, 1.07-1.09), and 6% among Hispanic individuals (RR, 1.06; 95% CI, 1.05-1.08), but remained steady among Asian/Pacific Islander individuals. The higher pace of growth among black individuals led to a widening black to white disparity of 3% per year (RR, 1.03; 95% CI, 1.01-1.05).

**Table 3.** Annual Changes in Legal Intervention Injuries per Population by Race or Ethnicity Among Males Aged 14 to 64 Years, From 2005 to 2015

Outcome	White	Black	Hispanic	Asian/Pacific Islander
**Injuries per 100 000 Population**				
Crude rates				
2005	55	159	66	11
2015	57	167	66	10
Rate ratio (95% CI)				
2005	1 [Reference]	2.90 (2.74-3.06)	1.23 (1.17-1.30)	0.21 (0.19-0.23)
2015	1 [Reference]	2.78 (2.64-2.93)	1.09 (1.04-1.15)	0.16 (0.14-0.18)
2005-2009				
Annual change by race/ethnicity	1.08 (1.07-1.09)	1.11 (1.09-1.13)	1.06 (1.05-1.08)	0.98 (0.94-1.02)
Difference in change across race/ethnicity	1 [Reference]	1.03 (1.01-1.05)	0.99 (0.96-1.00)	0.91 (0.87-0.94)
2009-2015				
Annual change by race/ethnicity	0.98 (0.97-0.99)	0.96 (0.95-0.97)	0.97 (0.97-0.98)	0.99 (0.97-1.01)
Difference in change across race/ethnicity	1 [Reference]	0.98 (0.97-0.99)	0.99 (0.98-1.00)	1.01 (0.99-1.04)
**Injuries per 100 000 Arrests**				
Crude rates				
2005	470	435	369	356
2015	566	623	565	508
Rate ratio (95% CI)				
2005	1 [Reference]	0.95 (0.91-1.00)	0.76 (0.74-0.79)	0.73 (0.66-0.81)
2015	1 [Reference]	1.11 (1.06-1.16)	1.00 (0.94-1.05)	0.90 (0.77-1.03)
2005-2013				
Annual change by race/ethnicity	1.07 (1.06-1.07)	1.07 (1.06-1.08)	1.08 (1.07-1.08)	1.02 (1.00-1.05)
Difference in change across race/ethnicity	1 [Reference]	1.00 (0.99-1.01)	1.01 (1.00-1.02)	0.96 (0.94-0.98)
2013-2015				
Annual change by race/ethnicity	0.89 (0.88-0.90)	0.94 (0.92-0.96)	0.96 (0.94-0.99)	1.10 (1.02-1.18)
Difference in change across race/ethnicity	1 [Reference]	1.06 (1.03-1.08)	1.08 (1.05-1.11)	1.23 (1.14-1.33)

During the remainder of the study period, from 2009 to 2015, rates began to decline across all groups, to nearly the levels seen in 2005. From 2009 to 2015, rates declined by 4% annually among black individuals (RR, 0.96; 95% CI, 0.95-0.97) and 2% among white individuals (RR, 0.98; 95% CI, 0.97-0.99), narrowing the racial disparity by 2% per year (RR, 0.98; 95% CI, 0.97-0.99). Rates also declined by 3% annually among Hispanic individuals (RR, 0.97; 95% CI, 0.97-0.98), but remained flat among Asian/Pacific Islander individuals.

By 2015, the end of the 11-year study period, disparities across groups approximated where they began in 2005. Rates among black individuals were 2.78-fold higher than white individuals (95% CI, 2.64-2.93), compared with 2.90-fold in 2005. Rates among Hispanic individuals were 1.09-fold higher than white individuals (95% CI, 1.04-1.15), down from 1.23-fold in 2005 (95% CI, 1.17-1.30).

**Figure 2.**
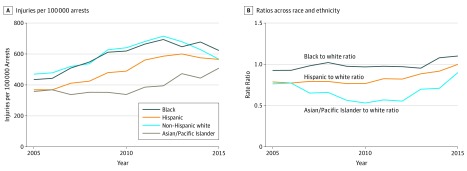
Legal Intervention Injuries per 100 000 Arrests and Ratios Across Race or Ethnicity of Males Aged 14 to 64 Years

In 2005, legal intervention injuries were highest among white individuals, at 470 per 100 000 arrest (Figure 2 and Table 3). The black to white ratio was nonsignificant (RR, 0.95; 95% CI, 0.91-1.00), while risk among Hispanic and Asian/Pacific Islander individuals were significantly lower as compared with white individuals (Hispanic to white RR, 0.76; 95% CI, 0.74-0.79; Asian/Pacific Islander to white RR, 0.73; 95% CI, 0.66-0.81). Over the 11-year study period, risk for all groups increased overall, although increases occurred from 2005 to 2013, after which risk began to decline for all but Asian/Pacific Islander individuals. From 2005 to 2013, annual proportional increases in risk were broadly similar across groups, with no changes in black to white or Hispanic to white ratios. However, the proportional declines experienced from 2013 to 2015 were greatest among white individuals, which in turn altered disparities. By 2015, risk among black individuals was 11% higher than white individuals (RR, 1.11; 95% CI, 1.06-1.16), and risk among Hispanic and Asian/Pacific Islander individuals was no longer significantly lower than white individuals.

Over the 11-year study period, legal intervention injuries per arrest increased more than legal intervention injuries per capita (Figure 1 and Figure 2). During this time period, the rate of legal intervention injuries per 100 000 population grew modestly (Figure 1). At the same time, the arrest rate per 100 000 declined substantially. Specifically, arrests of white individuals declined by 19%, black individuals by 28%, Hispanic individuals by 37%, and Asian/Pacific Islander individuals by 33%. An increase in injuries per population despite declining arrest rates necessarily leads to a significant increase in the likelihood of injury per arrest, which is reflected in the data.

## Discussion

Our findings suggest disparities in the rate of injury from legal intervention for different racial groups but do not identify the cause. One would expect that a group would have more injuries if that group was subject to more police interventions. Thus, we looked at legal intervention injuries per arrest for each group. Arrests do not account for all law enforcement actions (such as stop and frisk interactions short of arrest). As arrests decrease, stops without arrest could theoretically increase. That said, police use of force typically corresponds with stops that resulted in an arrest,^23^ so injuries per arrest can provide a rough proxy for the relevant legal interventions.

### Rate of Legal Intervention Injuries per Population and per Arrest

Our analysis of all ED visits in California from 2005 to 2014 coded for legal intervention injury points to several key findings. The data reveal proportional increases in the rate of legal intervention injury per population from 2005 to 2009 for white individuals, black individuals, and Hispanic individuals, and a widening black to white disparity. This was followed by proportional declines in rates from 2009 to 2015 and a narrowing disparity. Rates among Asian/Pacific Islander individuals were stable across the 11-year study period and substantially lower than other groups. Results are consistent with those of other investigators who found increases in legal intervention injury rates per population in the period from 2001 to 2014 based on national data.^18^

The current analysis also suggests that all groups experienced an increase in risk of injury per arrest, which may have resulted from declining arrest rates and changes in the offense mix of arrests. The greater focus on more severe offenses and fewer low-level arrests that resulted from the massive court-ordered reduction in the incarcerated population may have increased the risk of injury per arrest. Moreover, the decriminalization of marijuana possession in 2011 and the enactment of Senate Bill 18 in 2009 (which removed probation from certain offenders and reclassified certain property crimes as misdemeanors) redirected law enforcement to target more serious crimes, which could also have resulted in a greater likelihood of injury during arrest.

### Racial Differences in Legal Intervention Injuries per Population and per Arrest

Black individuals have much higher rates of injury per population than other groups, contributing to concerns about racial disparities in the police use of force. Our results indicate that much of the difference can be attributed to higher arrest rates for black individuals and therefore greater exposure to legal intervention. White and black individuals had similar risk of injury per arrest for much of the 11-year study period. Hispanic individuals had slightly fewer injuries per arrest than white individuals, and Asian/Pacific Islander individuals had much lower injuries per arrest. The absence of black to white disparities in injuries per arrest, at least through 2013, is supported by previous research.^17,24^ However, modest disparities do emerge at the end of the measurement period. In 2015, black individuals had a rate of injury per arrest of about 11% greater than the injury risk per arrest for white individuals. Differences may relate to the lower likelihood of arrests per stop among black individuals compared with white individuals, such that arrests underestimate police contact among black individuals to a greater degree.^17^ Growing disparities may also be a product of the larger reductions in arrests among black individuals during the study period. If police are decreasing arrests for minor offenses, and minor offenses carry the greatest discretion, the decline in arrests for low-level offenses would be expected to disproportionately affect minorities. As a result, the denominator for injuries per arrest would decline to the greatest extent among black individuals, while potentially becoming more concentrated on severe offenses.

The data highlight the importance of exploring the reasons for the racial disparities in arrest rates. Studies have highlighted various possible causes for those disparities, including differences in the rate of criminality, variations in community characteristics, and bias in arrest decisions.^25,26,27^ In addition, criminal justice policies that alter police priorities, change law enforcement strategies, or modify sentencing policies may be associated with the rate of injury per population by affecting the nature of police-public contacts or types of arrests. Additional research is warranted to explore the dynamics of the arrest disparities further.

### Severity of Legal Intervention Injuries Over the Past Decade

The proportion of injuries involving firearms or resulting in death has declined. Results may reflect evolving police agency procedures requiring an ED visit following use of force incidents, or jail policies requiring that individuals be examined prior to booking. Increasing use of tasers might have contributed to the corresponding decrease in firearm-related injuries. One converse result is notable: despite a decreased proportion of visits resulting in death, the share of visits that resulted in a hospital admission has been increasing.

The uptick in both mental health conditions and substance use disorders among patients with legal intervention injuries warrants further research. This trend may be a result of increases in hospital testing for these conditions, rather than a change in the population with legal intervention injuries, especially considering the parallel increases in these diagnoses for patients who have been assaulted. Alternatively, it could reflect changes in the populations police are interacting with in communities, following criminal justice reforms that shifted individuals from state prisons and paroles to county jails and probation.^28^ In either case, the data suggest there is a clear and potentially growing need for police agencies to continue to develop strategies for de-escalating interactions and proactively addressing mental health needs by collaborating with county mental health professionals.^29^

Policies and practices in policing are evolving, particularly as public concern about police use of force has increased in recent years. Our findings reveal variations in legal intervention injury rates per population across race or ethnicity, but far less variation in risk of injury on a per arrest basis. The results also suggest that injuries may be decreasing in severity. These findings highlight the value of using hospital data to assess the rate of nonfatal injuries, injuries that can have a profound impact on community health and affect the public’s trust in law enforcement. There is a need to promote the complete and accurate documentation of legal intervention injuries in EDs not only in California, but in other states as well.

### Limitations

The data set used offers a comprehensive look at ED visits in California, but faces certain limits. First, the results may have been affected by changes in hospital coding practices over time. Increased attention to use of force nationwide may have contributed to greater care among hospital staff to indicate law enforcement involvement in visits for legal intervention injury, inflating upward trends in injuries. Overall, injuries may be underestimated since legal intervention is not a valid cause code for all types of injuries that may occur in the course of police interaction, such as bites by police dogs or falls.

Second, we are unable to determine who, among those injured by police, seeks care in hospitals or whether this varies by race or ethnicity. Of those who do seek care, there could be differences across race or ethnicity in reporting to the clinician that police inflicted the injury, which is a prerequisite for coding legal intervention.

With regard to race or ethnicity, there may also be discrepancies in self-reported race or ethnicity used in population denominators and race or ethnicity selected in hospital visit records or arrests. Research comparing medical administrative data and self-reported survey responses has indicated alignment between those identifying as black or white, but underrepresentation in administrative data among those identifying as Hispanic or Asian/Pacific Islander.^30^ This would suggest the possibility that rates of injury per population could be underestimated in these 2 groups. Racial or ethnic categories are further limited by the absence of multiracial options.

While any instance of injury should be of concern, there is no way to determine from our data whether instances of force used were considered excessive force. Making such a determination involves a somewhat subjective judgment. Fatal injuries are likely to be underrepresented in the data if they occur without a hospital visit. The Counted reports there were 211 people killed by police in California in 2015.^14^

## Conclusions

Going forward, the recent shift to *ICD-10-CM* coding provides the ability to distinguish between legal intervention injuries for suspected lawbreakers, law enforcement officers, and bystanders. At the same time, efforts will be needed to ensure complete and accurate reporting by hospitals of legal intervention corresponding to injuries, to maximize the use of this data source.
